# The Impact of *pfmdr1* Gene Copy Number on the Efficacy of Dihydroartemisinin–Piperaquine in Treating Malaria in West Sumba and Kupang Districts, East Nusa Tenggara, Indonesia

**DOI:** 10.1155/japr/1352732

**Published:** 2026-05-20

**Authors:** Irdayanti Irdayanti, Hijral Aswad, Puji B. S. Asih, Ismail E. Rozi, Herdiana Herdiana, Ajib Diptyanusa, Helen D. Prameswari, Yenni Yusuf, Bahrani Bahrani, Najdah Hidayah, I. Made Artika, Din Syafruddin

**Affiliations:** ^1^ Master Program, Department of Biochemistry, Faculty of Mathematics and Natural Science, IPB University, Bogor, Indonesia, ipb.ac.id; ^2^ Hasanuddin University Medical Research Center, Makassar, Indonesia; ^3^ Eijkman Research Center for Molecular Biology, National Research and Innovation Agency, Cibinong, Indonesia, brin.go.id; ^4^ World Health Organization, Country Office for Indonesia, Jakarta, Indonesia, who.int; ^5^ Center for Online Health, Medical School, Faculty of Health, Medicine and Behavioral Sciences, The University of Queensland, Brisbane, Queensland, Australia, uq.edu.au; ^6^ Malaria Teamwork, Ministry of Health, Jakarta, Indonesia, behdasht.gov.ir; ^7^ Department of Parasitology, Faculty of Medicine, Hasanuddin University, Makassar, Indonesia, unhas.ac.id; ^8^ Biomedical Science Study Program, Graduate School of Hasanuddin University, Makassar, Indonesia; ^9^ Department of Biochemistry, Faculty of Mathematics and Natural Sciences, IPB University, Bogor, Indonesia, ipb.ac.id

**Keywords:** copy number variation, dihydroartemisinin–piperaquine (DHA-PPQ), *Plasmodium falciparum*, *Plasmodium falciparum multidrug resistance-1* (*Pfmdr1*)

## Abstract

The efficacy of antimalarial treatment, particularly artemisinin‐based combination therapy (ACT), continues to face persistent threats due to the development of drug resistance in *Plasmodium falciparum*. Although artemisinin derivatives remain effective, ACT regimen failure often results from decreased efficacy of the companion drug. Therefore, robust molecular surveillance focusing on genetic markers associated with companion drug resistance is essential to maintain global efficacy. The combination of dihydroartemisinin–piperaquine (DHA‐PPQ) has long been used as first‐line therapy and has proven effective; however, reports of resistance to piperaquine (PPQ) in Southeast Asia are cause for concern. The study is aimed at evaluating copy number variation (CNV) of the *Plasmodium falciparum multidrug resistance-1* (*Pfmdr1*) gene as part of a descriptive molecular surveillance approach in East Nusa Tenggara, Indonesia. A total of 41 dried blood spot (DBS) samples from patients with *falciparum* malaria treated with DHA‐PPQ were analyzed. The DNA extraction was performed using the QIAamp DNA Mini Kit, and CNV of the *Pfmdr1* gene was determined by real‐time PCR. The phenotypes of all 41 patients showed a good treatment response, with 100% adequate clinical and parasitological response (ACPR) on Day 42. Analysis of the *Pfmdr1* gene CNV identified 36 isolates (87.81%) with *a single copy* and 5 isolates (12.19%) with *multiple copies.* These findings indicate genetic variation related to selective pressure against PPQ. While DHA‐PPQ remains clinically effective in East Nusa Tenggara for now, the presence of these genetic markers necessitates a proactive surveillance strategy to detect shifts in drug sensitivity before widespread clinical failure occurs.

## 1. Introduction

Malaria remains a health problem in Indonesia, especially in eastern areas like Papua, even though many regions have achieved elimination. As of November 2025, there were 418,546 malaria cases nationwide, with 89% originating in Papua Province. The government is aimed at achieving national malaria elimination by 2030 through various strategies, including prevention, treatment, and systems. Data from the Indonesian Ministry of Health in 2024 shows that *Plasmodium falciparum* continues to be the primary cause of malaria cases, responsible for over half (50%–60%) of cases, particularly in moderate to high endemic areas such as Papua and East Nusa Tenggara provinces [1]. Since its introduction in 2010 as the first‐line treatment for malaria, dihydroartemisinin–piperaquine (DHA‐PPQ) has been shown to cure more than 98% of cases [2, 3]. The success of DHA‐PPQ as an artemisinin‐based combination therapy (ACT) depends on the mechanisms of its two drug components. Artemisinin acts quickly to treat malaria, while piperaquine (PPQ) has a long half‐life, providing extended protection [4, 5]. However, recent reports from several Southeast Asian countries of PPQ resistance suggest that the effectiveness of DHA‐PPQ may be endangered by drug selection pressure [6, 7].

The first cases of resistance to PPQ were reported in Cambodia in 2008–2010 and subsequently spread throughout the Greater Mekong Subregion (GMS) [7, 8]. Resistance to PPQ is clinically characterized by an increase in late treatment failure (LTF) and, at the molecular level, by amplification of the *Plasmepsin-2/3* (*Pfpm2-3*) gene and single‐nucleotide polymorphisms (SNPs) in the *Plasmodium falciparum chloroquine resistance transporter* (*Pfcrt*) gene [9]. In addition, decreased sensitivity to PPQ can also be associated with copy number variation (CNV) of the *Plasmodium falciparum multidrug resistance-1* (*Pfmdr1*) gene. The *Pfmdr1* gene is located on Chromosome 5, encodes a 12‐transmembrane‐domain protein, and is also known as *Plasmodium falciparum* P‐glycoprotein homolog‐1 (Pgh‐1). The *Pfmdr1* is found in the parasite’s digestive vacuole (DV), where chloroquine (CQ)‐ and quinoline (QN)‐based drugs act. Thus, CNV in the *Pfmdr1* gene can reduce PPQ accumulation in the parasite’s DV [10, 11].

In 2023, *a* therapeutic efficacy study (TES) of the antimalarial drug DHA‐PPQ was conducted in the West Sumba and Kupang Districts of East Nusa Tenggara Province, Indonesia. The study reported that 41 samples were examined and showed that all samples achieved adequate clinical and parasitological response (ACPR) on Day 42, and no SNPs were found in the *Plasmodium falciparum Kelch 13* (*Pfk13*) or *Pfcrt* genes. However, multiple copies of the *Pfpm2* gene are a critical mechanism of adaptive drug resistance [12]. These findings emphasize the importance of genetic monitoring, including *Pfmdr1* gene CNV analysis, to understand drug resistance in the region. This study is aimed at analyzing CNVs in the *Pfmdr1* gene in *P. falciparum* isolates from East Nusa Tenggara. This study was designed as a descriptive molecular surveillance to provide baseline data on genetic markers associated with PPQ resistance in Indonesia, where data linking molecular markers with phenotypic drug susceptibility remain limited.

## 2. Materials and Methods

### 2.1. Ethics

The samples used for molecular analysis were part of a follow‐up study to the “Therapeutic Efficacy Study (TES) for First Line of Antimalarial Drug Dihydroartemisinin‐Piperaquine/DHP‐PPQ in two sentinel sites in East Nusa Tenggara Province, Indonesia.” This study has obtained ethics approval from the Health Research Ethics Committee of the Faculty of Medicine, Hasanuddin University (No. 503/UN4.6.4.5.31/PP36/2022) and the National Research and Innovation Agency, Jakarta, Indonesia (No. 140/KE.03/SK/07/2024).

### 2.2. Study Site

**Figure 1:**
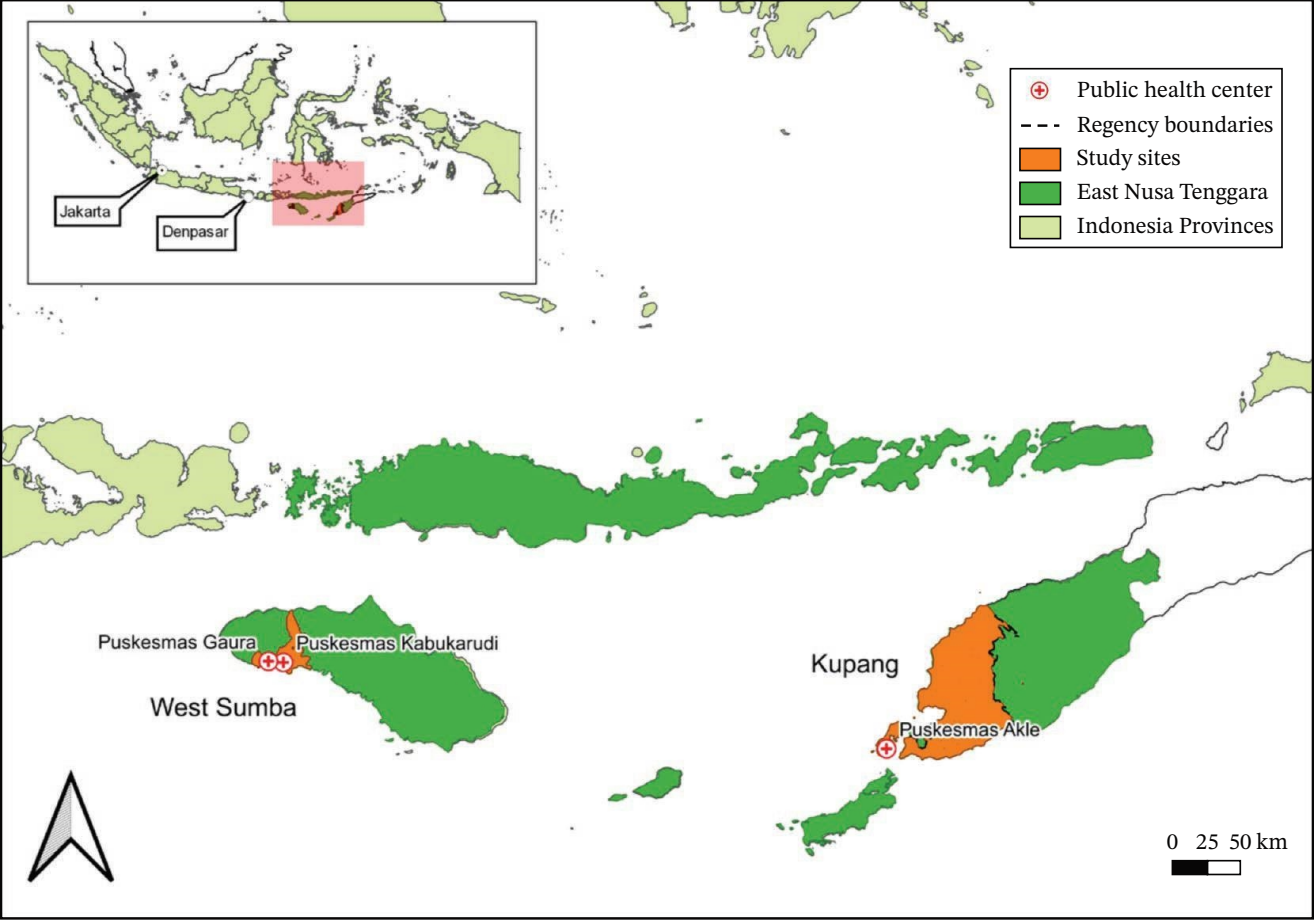
Sample collection locations.

The research location and sample collection (Figure 1) in this study were conducted in two areas with moderate malaria endemicity, namely, West Sumba District (Kabukarudi and Gaura primary health centers [PHCs]) and Kupang (Akle PHC), East Nusa Tenggara Province. The dried blood spot (DBS) samples from uncomplicated *P. falciparum* malaria patients enrolled in the DHA‐PPQ antimalarial drug trial in 2023 in West Sumba (*n* = 36, 1 participant lost to follow‐up, *n* = 35) and Kupang (*n* = 6), East Nusa Tenggara Province, were used to analyze the molecular profile of resistance to *P. falciparum*. All participants completed the 42‐day follow‐up period. The treatment efficacy results for all participants were classified as ACPR, with a 100% cure rate at both study sites, as detailed in a previous study [12].

### 2.3. DNA Extraction

The samples used were obtained from the TES of the antimalarial drug DHA‐PPQ conducted in East Nusa Tenggara Province in 2023. Following the method of Chenet et al., DNA was extracted from DBS samples using the QIAamp DNA Mini Kit (Qiagen) [13]. The extracted DNA was then stored at −20°C until used for molecular analysis. This process was carried out to ensure that the DNA quality was sufficient for target gene analysis.

### 2.4. Real‐Time PCR Detection of CNV of the *Pfmdr1* Gene

CNV analysis of the *Pfmdr1* gene was performed using a probe‐based real‐time PCR method, as previously reported [9, 14]. The primer sequences used are shown in Table 1. The total PCR reaction used was 20 *μ*L, consisting of Thunderbird Sybr Mix (Toyobo), *Pfmdr1-specific* primers, *β*‐tubulin reference gene primers, probe primers, ddH_2_O, and DNA template. Each sample was run in duplicate, with strain 3D7 used as a positive control. PCR conditions included 95°C for 30 s, followed by 40 cycles of 95°C for 5 s and 58°C for 10 s, then a melt curve from 65°C to 95°C.

**Table 1:** Primer sequences for *Pfmdr1* gene *copy number* detection [13].

No.	Primer name	Primer sequence
1.	PfMDR1‐1F	TGCATCTATAAAACGATCAGACAAA
2.	PfMDR1‐1R	TCGTGTGTTCCATGTGACTGT
3.	PfMDR1 probe	FAM‐TTTAATAACCCTGATCGAAATGGAACCTTTG‐TAMRA
4.	PFB‐tubulin‐3F	AAAAATATGTGCGCAAGTGA
5.	PFB‐tubulin‐3R	AACTTCCTTTGTGGACATTCTTCCT
6.	PFB‐tubulin‐probe	VIC‐TAGCACATGCCGTTAAATATCTTCCATGTCT‐TAMRA

### 2.5. Analysis

Analysis of real‐time PCR results using Microsoft Excel to determine copy number by entering the threshold value (Ct) of the target gene (*Pfmdr1*) and reference gene (*β*‐tubulin) using the 2^−*ΔΔ*Ct^ method. Samples with values close to 1 are considered to have a *single copy*, while values > 1.5 are categorized as multiple copies [15]. The Wilson score interval is used to estimate a confidence interval for proportions in multiple copies.

## 3. Results

**Figure 2:**
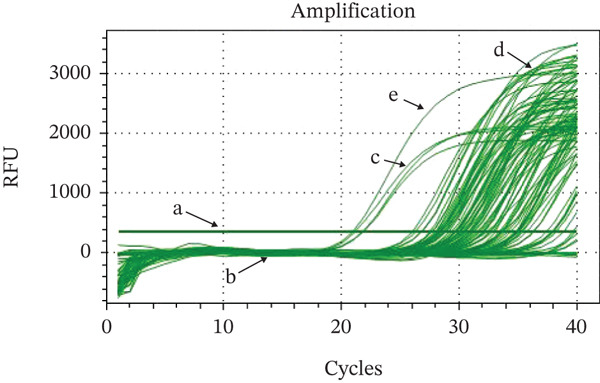
Real‐time PCR amplification results for the Pfmdr1 gene: (a) threshold, (b) negative control, (c) single copy number, (d) multiple copies number, and (e) tubulin gene.

CNV analysis of the *Pfmdr1* gene using *real-time* PCR was successfully performed on all samples (Figure 2). A total of 5 of 41 samples amplified for the *Pfmdr1* gene showed > 1.5 copies (12.19%), indicating multiple copies, while the other 36 samples (87.81%) had a single copy (Table 2). At a 95% confidence level, the confidence interval for multiple copies is 0.122 ± 0.101 (0.0532–0.255).

**Table 2:** Distribution of the *Pfmdr1* gene CNV.

Category	Number of samples (*n*)	Percentage (%)
Single copy (< 1.5)	36	87.81
Multiple copies (> 1.5)	5	12.19

## 4. Discussion

This study is a follow‐up molecular analysis of the TES of the antimalarial drug DHA‐PPQ conducted in West Sumba and Kupang Districts, East Nusa Tenggara Province, in 2023. DBS samples from uncomplicated *falciparum* malaria patients enrolled in the DHA‐PPQ antimalarial drug trial in 2023 in West Sumba District (*n* = 36, 1 participant lost to follow‐up, *n* = 35) and Kupang (*n* = 6), East Nusa Tenggara Province, were used to analyze the molecular profile of resistance to *P. falciparum*. All participants completed the 42‐day follow‐up period. The treatment efficacy results for all participants were classified as ACPR, with a 100% cure rate at both study sites, as detailed in a previous study [12]. The study also found multiple copies of the *Pfpm2* gene in 10 out of 41 samples (24.39%).

Amplification of the *Pfmdr1* gene using real‐time PCR found that 36 samples (87.81%) had a single copy, while 5 samples (12.19%) had multiple copies of the *Pfmdr1* gene. These results indicate the potential for PPQ selection pressure in the region. Increased CNV *of the Pfmdr1* gene is associated with decreased parasite sensitivity to several antimalarial drugs, particularly quinoline‐based agents such as mefloquine, lumefantrine, and PPQ [16, 17]. This is due to increased expression of the PfMDR1 transporter, which can affect drug accumulation within the parasite’s DV [18]. However, the low prevalence of CNVs in *Pfmdr1* is consistent with another study of *P. falciparum* populations in Keerom [19], in which *Pfmdr1* amplification was also low but still present.

The results of research in West Sumba and Kupang Districts, when compared to other regions in Southeast Asia, show that multiple CNVs of the *Pfmdr1* gene are relatively low (12.19%). Research in Cambodia and Thailand reports a higher multiple of CNV, around 50%–60%, especially in areas with a history of widespread use of mefloquine or PPQ [8, 17]. These differences are attributed to variations in drug selection pressure, malaria transmission intensity, and parasite migration patterns between regions [20]. The number of multiple copy numbers identified in this study remains relatively low, but it warrants attention because several studies have shown that *Pfmdr1* gene CNVs are associated with resistance to PPQ, especially when accompanied by mutations in other genes, such as *Pfcrt* or *Pfpm2* [7, 9]. Therefore, continuous molecular surveillance is needed to detect early signs of resistance to DHA‐PPQ in the West Sumba and Kupang Districts, given that DHA‐PPQ has been used for more than a decade as a first‐line treatment in Indonesia. This study has several limitations, particularly the relatively small sample size (*n* = 41), with only 6 samples originating from Kupang. This study acknowledges that the study’s sample size was not achieved due to the ongoing decline in malaria incidence at the study site, particularly in Kupang. Although some malaria cases were identified during the study, many were ineligible due to very low parasite densities. In addition, the limitations stem from the small number of eligible participants enrolled in the TES during the study period, as well as strict inclusion criteria and a 42‐day follow‐up requirement. These factors reduced the availability of complete and high‐quality samples for molecular analysis. Therefore, further research with a broader coverage area and a larger sample size is needed to monitor resistance more effectively.

This study was designed as a molecular surveillance study using samples derived from a TES, with a primary focus on clinical outcomes, ACPR. Molecular markers such as *Pfmdr1* CNV are widely used as early indicators of potential drug resistance and provide valuable insights, especially in settings where in vitro assays are not routinely feasible. Future studies integrating molecular markers with in vitro or ex vivo drug susceptibility assays are needed to better understand the functional impact of *Pfmdr1* amplification on PPQ resistance in Indonesia.

## 5. Conclusion

This study shows that DHA‐PPQ treatment remains highly effective against uncomplicated *P. falciparum* malaria in West Sumba and Kupang Districts, with a 100% cure rate and no treatment failures. Molecularly, 5 of 41 samples (12.19%) showed multiple CNVs in the *Pfmdr1* gene. Although low, the presence of multiple CNVs *in the Pfmdr1* gene may represent an early stage of selection pressure against PPQ and warrants regular monitoring.

## Funding

The study was funded by the World Health Organization (10.13039/100004423, TSA 203033242) and Badan Riset dan Inovasi Nasional (10.13039/100020473).

## Conflicts of Interest

The authors declare no conflicts of interest.

## Data Availability

Data sharing is not applicable to this article as no datasets were generated or analyzed during the current study.

## References

[1] Indonesian Ministry of Health , Malaria Cases in Indonesia, 2025, https://malaria.kemkes.go.id/case.

[2] Poespoprodjo J. , Kenangalem E. , Wafom J. , Chandrawati F. , Puspitasari A. , Ley B. , Trianty L. , Korten Z. , Surya A. , Syafruddin D. , Anstey N. , Marfurt J. , Noviyanti R. , and Price R. , Therapeutic Response to Dihydroartemisinin–Piperaquine for *P. falciparum* and *P. vivax* Nine Years After Its Introduction in Southern Papua, Indonesia, American Journal of Tropical Medicine and Hygiene. (2018) 98, no. 3, 677–682, 10.4269/ajtmh.17-0662, 2-s2.0-85043519739, 29345221. 29345221 PMC5850981

[3] Indonesian Ministry of Health , Annual Malaria Report Directorate of P2P, 2022, 2022, https://malaria.kemkes.go.id/sites/default/files/2023-11/Annual%20Malaria%20Report%202022_English%20version%20%281%29.pdf.

[4] Popovici J. , Vantaux A. , Primault L. , Samreth R. , Piv E. P. , Bin S. , Kim S. , Lek D. , Serre D. , and Menard D. , Therapeutic and Transmission-Blocking Efficacy of Dihydroartemisinin/Piperaquine and Chloroquine Against *Plasmodium vivax* Malaria Cambodia, Emerging Infectious Diseases. (2018) 24, no. 8, 1516–1519, 10.3201/eid2408.170768, 2-s2.0-85050398698, 29798745. 29798745 PMC6056113

[5] World Health Organization , Antimalarial Drug Combination Therapy. Report of a WHO Technical Consultation 2021, 2025, https://iris.who.int/bitstream/handle/10665/66952/WHO_CDS_RBM_2001.3.pdf.

[6] Leang R. , Barrette A. , Bouth D. M. , Menard D. , Abdur R. , Duong S. , and Ringwald P. , Efficacy of Dihydroartemisinin-Piperaquine for Treatment of Uncomplicated *Plasmodium falciparum* and *Plasmodium vivax* in Cambodia, 2008 to 2010, Antimicrobial Agents and Chemotherapy. (2013) 57, no. 2, 818–826, 10.1128/AAC.00686-12, 2-s2.0-84872849978, 23208711. 23208711 PMC3553743

[7] Amato R. , Lim P. , Miotto O. , Amaratunga C. , Dek D. , Pearson R. , Garcia J. A. , Neal A. , Sreng S. , Suon S. , Drury E. , Jyothi D. , Stalker J. , Kwiatkowski D. , and Fairhurst R. , Genetic Markers Associated With Dihydroartemisinin–Piperaquine Failure in *Plasmodium falciparum* Malaria in Cambodia: A Genotype–Phenotype Association Study, Lancet Infectious Diseases. (2017) 17, no. 2, 164–173, 10.1016/S1473-3099(16)30409-1, 2-s2.0-85006176728, 27818095. 27818095 PMC5564489

[8] Imwong M. , Suwannasin K. , Kunasol C. , Sutawong K. , Mayxay M. , Rekol H. , Smithuis F. M. , Hlaing T. M. , Tun K. M. , Pluijm R. W. V. D. , Tripura R. , Miotto O. , Menard D. , Dhorda M. , Day N. P. J. , White N. J. , and Dondrop A. M. , The Spread of Artemisinin-Resistant *Plasmodium falciparum* in the Greater Mekong Subregion: A Molecular Epidemiology Observational Study, Lancet Infectious Diseases. (2017) 17, no. 5, 491–497, 10.1016/S1473-3099(17)30048-8, 2-s2.0-85011304306, 28161569. 28161569 PMC5406483

[9] Witkowski B. , Duru V. , Khim N. , Ross L. S. , Saintpierre B. , Beghain J. , Chy S. , Kim S. , Ke S. , Kloeung N. , Eam R. , Khean C. , Ken M. , Loch K. , Bouillon A. , Domergue A. , Ma L. , Bouchier C. , Leang R. , Huy R. , Nuel G. , Barale J. C. , Legrand E. , Ringwald P. , Fidock D. A. , Puijalon O. M. , Ariey F. , and Ménard D. , A Surrogate Marker of Piperaquine-Resistant *Plasmodium falciparum* Malaria: A Phenotype–Genotype Association Study, Lancet Infectious Diseases. (2017) 17, no. 2, 174–183, 10.1016/S1473-3099(16)30415-7, 2-s2.0-85006173671, 27818097. 27818097 PMC5266792

[10] Fidock D. A. , Nomura T. , Talley A. K. , Cooper R. A. , Dzekunov S. M. , Ferdig M. T. , Ursos L. M. B. , Sidhu A. S. , Naude B. , Deitsch K. W. , Su X. Z. , Wootton J. C. , Roepe P. D. , and Wellems T. E. , Mutations in the *P. falciparum* Digestive Vacuole Transmembrane Protein Pfcrt and Evidence for Their Role in Chloroquine Resistance, Molecular Cell. (2000) 6, no. 4, 861–871, 10.1016/S1097-2765(05)00077-8, 2-s2.0-0033636607, 11090624. 11090624 PMC2944663

[11] Veiga M. I. , Ferreira P. E. , Malmberg M. , Jornhagen L. , Bjorkman A. , Nosten F. , and Gil J. P. , pfmdr1 Amplification Is Related to Increased *Plasmodium falciparum* In Vitro Sensitivity to the Bisquinoline Piperaquine, Antimicrobial Agents and Chemotherapy. (2012) 56, no. 7, 3615–3619, 10.1128/AAC.06350-11, 2-s2.0-84862538598, 22508315. 22508315 PMC3393437

[12] Irdayanti , Aswad H. , Asih P. B. , Rozi I. E. , Basri H. H. , Diptyanusa A. , Prameswari H. D. , Yusuf Y. , Bahrani , Hidayah N. , Artika I. M. , and Syafruddin D. , Genetic Profiles of Plasmodium Falciparum Isolates From a Therapeutic Efficacy Study on the Antimalarial Drug Dihydroartemisinin-Piperaquine in the West Sumba and Kupang Districts of East Nusa Tenggara Province, Indonesia, BIO Web of Conferences. (2025) 184, 01018, 10.1051/bioconf/202518401018.

[13] Chenet S. , Okoth S. , Kelley J. , Lucchi N. , Huber C. , Vreden S. , Oliveira A. M. , Barnwell J. , Udhayakumar V. , and Adhin M. , Molecular Profile of Malaria Drug Resistance Markers of *Plasmodium falciparum* in Suriname, Antimicrobial Agents and Chemotherapy. (2017) 61, no. 7, e02655, 10.1128/AAC.02655-16, 2-s2.0-85021684104, 28438929. 28438929 PMC5487647

[14] Rahmasari F. V. , Asih P. B. S. , Rozi I. E. , Wangsamuda S. , Risandi R. , Dewayanti F. K. , Permana D. H. , Syahrani L. , Prameswari H. D. , Basri H. H. , Bustos M. D. G. , Charunwatthana P. , Dondorp A. M. , Imwonga M. , and Syafruddin D. , Evolution of Genetic Markers for Drug Resistance After the Introduction of Dihydroartemisinin–Piperaquine as First-Line Anti-Malarial Treatment for Uncomplicated falciparum Malaria in Indonesia, Malaria Journal. (2023) 22, no. 1, 10.1186/s12936-023-04658-4, 37553646. PMC10410932 37553646

[15] Ferreira I. D. , Rosario V. E. D. , and Cravo P. V. L. , Real-Time Quantitative PCR With SYBR Green I Detection for Estimating Copy Numbers of Nine Drug Resistance Candidate Genes in Plasmodium falciparum, Malaria Journal. (2006) 5, no. 1, 1–6, 10.1186/1475-2875-5-1, 2-s2.0-33646272412, 16420686. 16420686 PMC1363351

[16] Sidhu A. B. S. , Uhlemann A. C. , Valderramos S. G. , Valderramos J. C. , Krishna S. , and Fidock D. A. , Decreasing pfmdr1 Copy Number in *Plasmodium falciparum* Malaria Heightens Susceptibility to Mefloquine, Lumefantrine, Halofantrine, Quinine, and Artemisinin, Journal of Infectious Diseases. (2006) 194, no. 4, 528–535, 10.1086/507115, 2-s2.0-33746302565, 16845638. 16845638 PMC2978021

[17] Price R. N. , Cassar C. , Brockman A. , Duraisingh M. , van Vugt M. , White N. J. , Nosten F. , and Krishna S. , The pfmdr1 Gene Is Associated With a Multidrug-Resistant Phenotype in *Plasmodium falciparum* From the Western Border of Thailand, Antimicrobial Agents and Chemotherapy. (1999) 43, no. 12, 2943–2949, 10.1128/aac.43.12.2943, 10582887. 10582887 PMC89592

[18] Cowman A. F. , Galatis D. , and Thompson J. K. , Selection for Mefloquine Resistance in *Plasmodium falciparum* Is Linked to Amplification of the pfmdr1 Gene and Cross-Resistance to Halofantrine and Quinine, Proceedings of the National Academy of Sciences. (1994) 91, no. 3, 1143–1147, 10.1073/pnas.91.3.1143, 2-s2.0-0028014644, 8302844. PMC521470 8302844

[19] Rahmasari F. V. , Genetic Analysis of Malaria Parasite Isolates From Southwest Sumba and Keerom Districts, Indonesia, 2023, Mahidol University.

[20] Ariey F. , Witkowski B. , Amaratunga C. , Beghain J. , Langlois A. , Khim N. , Kim S. , Duru V. , Bouchier C. , Ma L. , Lim P. , Leang R. , Duong S. , Sreng S. , Suon S. , Chuor C. , Bout D. , Ménard S. , Rogers W. , Genton B. , Fandeur T. , Miotto O. , Ringwald P. , Bras J. L. , Berry A. , Barale J. , Fairhurst R. , Vical F. B. , Puijalon O. M. , and Ménard D. , A Molecular Marker of Artemisinin-Resistant *Plasmodium falciparum* Malaria, Nat.(2014) 505, no. 7481, 50–55, 10.1038/nature12876, 2-s2.0-84892372929, 24352242. PMC5007947 24352242

